# Sulfate-Reducing Bacteria as an Effective Tool for Sustainable Acid Mine Bioremediation

**DOI:** 10.3389/fmicb.2018.01986

**Published:** 2018-08-22

**Authors:** Ayansina S. Ayangbenro, Oluwaseyi S. Olanrewaju, Olubukola O. Babalola

**Affiliations:** Food Security and Safety Niche, Faculty of Natural and Agricultural Sciences, North-West University, Mmabatho, South Africa

**Keywords:** bioleaching, heavy metals, microorganism, mine wastes, mining, tailings

## Abstract

Mining industries produce vast waste streams that pose severe environmental pollution challenge. Conventional techniques of treatment are usually inefficient and unsustainable. Biological technique employing the use of microorganisms is a competitive alternative to treat mine wastes and recover toxic heavy metals. Microorganisms are used to detoxify, extract or sequester pollutants from mine waste. Sulfate-reducing microorganisms play a vital role in the control and treatment of mine waste, generating alkalinity and neutralizing the acidic waste. The design of engineered sulfate-reducing bacteria (SRB) consortia will be an effective tool in optimizing degradation of acid mine tailings waste in industrial processes. The understanding of the complex functions of SRB consortia vis-à-vis the metabolic and physiological properties in industrial applications and their roles in interspecies interactions are discussed.

## Introduction

Urbanization and increase in the world’s population, with an attendant rise in energy and mineral demands, have continued to drive mining activities. The goal of the mining industry is to meet the requirements for energy and mineral resources, enhance infrastructural development and quality of life of the populace. While mining and mineral extraction have contributed significantly to the advancement of human civilization and national economies, they have also caused serious environmental degradation. Mineral extraction and its resultant need for the disposal of wastes, slurry and water can have major environmental implications (Jain et al., 2016).

**FIGURE 1:**
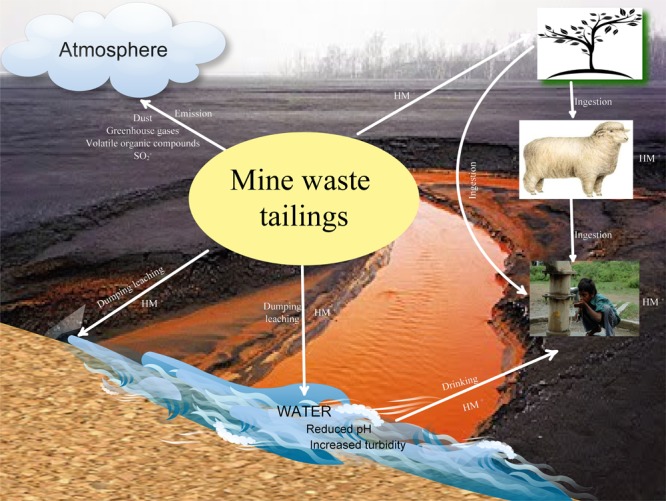
Destruction of the ecosystem by mine wastes.

Mining and mineral processing activities generate massive amounts of toxic, corrosive, or flammable materials. Mining sites are surrounded by stockpiles of waste dumps, tailings ponds and processing chemicals. Mine wastes are by-products of mining operations which have no economic value. They consist of ash, flue dust, gangue, industrial minerals, loose sediment, metals, metallurgical slag and wastes, mill tailings, mineral fuels, ore, particulate emissions, processing chemicals and fluids, roasted ore and rock. It has been established that at least a ton of mining waste is generated for every ton of metal ore extracted. These waste streams demand informed planning and decision-making in matters of waste reduction, resource recovery, waste disposal and environmental protection (Hudson-Edwards et al., 2011). The release of these wastes into the environment can have significant impact on surface water, groundwater, air and land resources (**Figure 1**).

Striking features of mining industries are the incidents of poor waste management. Dam failures, seepages, tailings spills, unrehabilitated sites and instances of direct disposal into waterways can result in serious and long-term environmental consequences (Franks et al., 2011). The large quantity of waste streams generated destroys the landscape, disrupt ecosystems including flora and fauna. Waste materials that remain after extraction of usable ores are dumped on the surrounding land, which is the source of toxic metals. This leaves the land without topsoil, nutrients and vegetation (Mukhopadhyay and Maiti, 2010; Tripathi et al., 2016). Mine wastes do not encourage plant growth and microbial populations playing key functional roles in soil due to acidic pH, lack of adequate soil structure, small particle size, high metal content, limited organic matter and quantities of essential nutrients (Valentín-Vargas et al., 2014).

Tailings are processed waste with finely ground rock that varies in size, from which valuable materials have been extracted. They consist of the materials formed after the enrichment process of the ore and are normally stored in impoundments. They result from oxidation of sulfide minerals which are likely to produce acid mine. Consequently, acidification increases the dissolution of toxic heavy metals from open pits and tailings. Mine tailings contain high concentrations of toxic metal which contaminates land and water ecosystem (Tripathi et al., 2016). These toxic metals are non-degradable, persist in the ecosystem and may pollute surface and underground water resources, thus, posing a serious public health challenge (Ayangbenro and Babalola, 2017).

There has been growing concern about the fate of tailings and the consequent release of environmental pollutants from dust, failure of dam wall, tailings dam seepage, or direct discharge of mine tailings into waterways. Therefore, the management of mine waste to meet the challenge of sustainable development amongst the various stakeholders concerned is paramount (Franks et al., 2011).

Bioremediation is an innovative technique for the treatment of acid mine tailings waste (AMTW) polluted environments which involves using living organisms (microorganisms and plants) to reduce the effects of acid mine tailings into less harmful forms. This method is an appealing alternative to conventional methods because of its cost effectiveness, maintaining ecological balance and aids in reestablishing polluted environments (Martins et al., 2011). Bioremediation processes can function naturally or be enhanced by supplementing the process with electron acceptor, nutrient or other factors (Lin and Lin, 2005). Sulfate-reducing bacteria (SRB) facilitate the conversion of sulfate to sulfide with the sulfides reacting with heavy metals to precipitate toxic metals as metal sulfide. These metal sulfides are stable and can easily be removed from AMTW (Cohen, 2006). This process facilitates the removal of toxic metals from AMTW by SRB. SRB thrive in anaerobic environment with pH range of 5–8. These conditions are essential for optimum removal of metal and sulfate from tailing wastes.

Generation of AMTW and its attendant effects on microorganisms are dealt with in this paper. Bioremediation is proposed as an effective tool for removal of AMTW by SRB and the potential of SRB consortia for effective and sustainable remediation process.

## Acid Mine Tailings Waste Generation

Acid mine tailings wastes comprise the different minerals found in the mined rock. In the un-mined rock, located deep beneath the soil, the reactive minerals are sheltered from chemical oxidation. In the absence of oxygen, the sulfide minerals have low chemical solubility and are stable in that state. However, upon excavation and exposure to atmospheric oxygen and water, there begins a series of bio-geochemical processes that can lead to acid mine production. Naturally occurring bacteria can also play active role in accelerating acid mine generation by promoting the breakdown of sulfide minerals, hence oxidation of the sulfide minerals. The process results in low pH which is characterized by high heavy metal concentrations. Factors that are responsible for the rate of acid generation in AMTW are degree of saturation with water; oxygen, pH, temperature, chemical activity of Fe^3+^, chemical activation energy required to begin acid generation, sulfide minerals, surface area of exposed metal sulfide, and iron oxidizing bacteria (Akcil and Koldas, 2006).

Most metals are associated with pyrite (the most abundant sulfide mineral on the planet) and occur mainly as sulfide ores. Metal sulfides are reduced to ferrous on reaction with pyrite by ferric iron (the main oxidant). This reaction is independent of oxygen and is the most important step in the oxidation of sulfide minerals. Ferrous iron oxidation can be mediated biologically (by iron-oxidizing bacteria such as *Gallionella ferruginea*) or chemically by molecular oxygen at pH above 4. Ferrous iron chemical oxidation rate is negligible below pH 4, thus the activities of acidophilic iron-oxidizing bacteria play a crucial role in acid mine drainage generation (Hallberg, 2010).

Sulfide oxidation and acid production in mining waste can be hindered by acid consuming processes in varying degrees, depending on the availability of acid consuming minerals. Carbonates (such as calcite and dolomite) and silicates can neutralize the effect of the low pH when present in mine tailings waste, thus, liberating heavy metals and other elements into solution and preventing them from been transported to the surrounding environment to any considerable amount. At low pH, heavy metals are stable in solution and mobile, and has pH increases, they become adsorbed and thus become immobile (Dold, 2014).

The chemical composition of AMTW is determined by the minerals, microbiological, chemical and physical properties of the mining site. The site’s physical properties include the density, size and source of the waste material as well as the hydrological properties of the site. The type of minerals that are mined (metals, metalloids or coal), could also influence acid composition. Chemical composition includes elevated concentrations of different kinds of metals. Dissolved salts, other than sulfates, may also be present (Kuyucak, 2002).

## Effects of Acid Mine Tailings Waste on Microorganisms

Toxicity of AMTW is caused primarily by acidic pH and heavy metal content. The synergistic effect between acidic pH and heavy metals increases the bioavailability of heavy metals, thus, increasing biotoxicity. Exposure to heavy metals from mine waste can be linked to many disorders and diseases (Ayangbenro and Babalola, 2017).

Acid mine tailings causes alteration and destruction of ecosystem, landscape deterioration, loss of biodiversity and buildup of pollutants in the environment. The resulting effect is the reduction and destruction of physical habitats for microorganisms, as well as nutritional, ecological and evolutionary problems (Ayangbenro and Babalola, 2017), fragmentation of habitat and intrusion by feral organisms and weed species (Jain et al., 2016). This also has dire consequences on the balance of the ecosystem and its resultant disruption of the food web, thus having negative impacts on the lifestyles of aboriginal organisms.

Exposure to unfavorable conditions, such as toxic metals and metalloids and low pH in AMTW, induces stress responses, exhibiting characteristic changes in microbial cell morphology and assembly (Chakravarty and Banerjee, 2008). These changes inhibit cell growth. Also, microbial enzymes are denatured by acid, which stops metabolic functions. This results in loss of building blocks of macromolecules that are essential for growth. The hydronium ions of rain also mobilize toxins such as aluminum, causing leaching of important nutrients and minerals such as magnesium that are required for growth (Davies and Mundalamo, 2010). Furthermore, those organisms that are unable to tolerate low pH changes are destroyed, resulting in loss of diversity.

Acid mine tailings wastes only supports heterotrophic microbial communities that are severely stressed. Consequently, carbon utilization diversity and species richness are extremely low among the microbial communities compared with unpolluted environment. Microbial communities that dominate in such environments are the iron- and sulfur-oxidizing autotrophs (Mendez and Maier, 2008). Changes in microbial community structure and activity is also affected by AMTW. This also results in significant reduction in soil nutrient turnover because of the combined effect of H^+^ and Al^3+^ (Sahoo et al., 2010).

The production of reactive oxygen species, due to heavy metal toxicity, damages the cell plasma membrane. The damage to the plasma membrane may include alteration of biophysical parameters, increase in membrane permeability, loss of intracellular ions or enhanced accumulation of extracellular ions. Changes in the activities of essential membrane-bound enzymes, whose functions are dependent on membrane-bound lipids, are also affected (Poljsak et al., 2011).

Other effects of heavy metal ions from acid mine tailings on microorganisms include apoptosis, ATP synthesis inhibition, damage of nucleic acid, denaturation of protein, displacement of an essential metal, induction of oxidative stress, interruption of the function of important proteins regulating fundamental processes including growth, impairment of DNA repair, inhibition of cell division and transcription (Wysocki and Tamás, 2011; Fashola et al., 2016).

## Bioremediation of Acid Mine Tailings Waste

Acid mine is typically prevented by the introduction of alkaline materials to sulfide-rich mine wastes. The process is a chemical-neutralizing treatment that promotes neutralization of acid, reduced solubility of metals and consequent retention of metals in solution by precipitation. The rate of ferrous iron oxidation in AMTW is accelerated by the addition of alkaline materials. This also increases the pH and causes metal precipitation in solution as hydroxides and carbonates. However, these conventional treatment processes (alkaline addition, chemical precipitation, ion exchange and fluidized bed-ion) require significant financial resources in terms of capital and operating costs. Most of these processes often involve using chemicals that can result in pollution and requiring additional cleanup (Hilson and Murck, 2001), and some of these techniques are not sustainable. This makes the search for low cost treatment with better contaminant removal an alternative. Presently, the cost-effective and environmentally friendly technique for treating AMTW is through the use of plants and microorganisms, though in some cases it has not been successful due to location and/or technological limitations. In some cases, biological reactors may require a constant biomass supply feed to operate and if absent, such reactors will not function (Hilson and Murck, 2001).

Bioremediation is considered as an alternative and economically viable technique compared to conventional remediation techniques, and sulfate-reducing microorganisms have been applied for the treatment of acid mine waste. Due to simultaneous elimination of sulfate and metals, and the possibility to reuse, bioreactors with SRB represent a promising option for remediation of AMTW. Nonetheless, the efficiency of SRB depend on various factors such as microbial diversity, type of carbon source, and reactor configuration (Martins et al., 2011). Microbial remediation with SRB is sustainable with regard to the operating cost of neutralizing the acidic effect, buffering of AMTW and heavy metal removal (Anawar, 2015).

The basis of microbial remediation of AMTW is to enhance the capabilities of microbes to neutralize acidity and immobilize metals. Thus, the reactions generating AMTW are being reversed (Johnson and Hallberg, 2005; Pérez-López et al., 2007; Sánchez-Andrea et al., 2014). Alkalinity production by microbial groups mostly involves reduction reactions that include ammonification, denitrification, iron and manganese reduction, methanogenesis and sulfate reduction (Johnson and Hallberg, 2005). Harnessing the acid-consuming potential of these microbial groups ameliorates the damaging effects of these waste streams before discharge into the environment.

Acid mine tailings usually have low concentrations of organic carbon, thus the need for the addition of electron donor (molecular hydrogen or organic compounds) is required to enhance microbial activity. Autotrophic iron-oxidizing acidophiles carry out oxidation reactions using inorganic carbon, while the reduction processes are stimulated by organic carbon as carbon source and electron donor (Hallberg, 2010; Sánchez-Andrea et al., 2014). The treatment process can be applied *in situ* or *ex situ. In situ* technique involves the use of anaerobic wetlands and permeable reactive barriers while *ex situ* uses compost bioreactors or sulfidogenic reactors. *In situ* technologies are durable, clean and cheap and are considered the preferable option to *ex situ* techniques (Sánchez-Andrea et al., 2014). The treatment procedure needs some level of sophistication to ensure that effluents meet the required standard. This sophistication is determined by certain factors that take into account the chemical and sludge characteristics of the mine tailings, local climate and terrain, quantity of water needed for treatment and the projected life of the treatment plant (Akcil and Koldas, 2006).

### Remediation by Sulfate-Reducing Bacteria

Sulfate-reducing bacteria can be used to remediate acid mine tailings making use of the oxygen and enriched carbon source produced by algae (Hilson and Murck, 2001; Hallberg, 2010). They use sulfate as a terminal electron acceptor with SO_4_^2-^ being converted into H_2_S. The isolation of acid tolerant SRB, which catalyze the reduction of sulfate to sulfide that transforms sulfuric acid to hydrogen sulfide and generates alkalinity, has allowed the development of innovative treatment techniques. This reductive reaction is a rate limiting procedure for selectively removing toxic metals from acid mine tailings since many of them form highly insoluble sulfides. Metal recovery is achieved by regulating the concentration of the reactant sulfate through pH control in the bioreactor (Johnson and Hallberg, 2005; Hallberg, 2010). Toxic metals are thus effectively removed from mine tailings wastes, and the wastes are changed into useful products. The advantage of bioremediation is that it has low maintenance costs, coupled with the fact that the solid-phase products of water treatment are retained within the wetland sediments (Johnson and Hallberg, 2005).

Sulfide oxidation and reduction of mineral oxides within the exposed mine tailings can result in mobilization of metals previously bound within the mineral matrix by SRB present in mine tailings. These SRB play significant roles in mobilization and elimination of toxic metals through dissolution and precipitation, and recovery of valuable metals from the low-grade ores. Microorganisms that have been employed in microbial leaching of metals from ores/waste span different genera and include bacteria such as *Acidiphilium cryptum, Acidithiobacillus ferrooxidans, At. caldus, At. thiooxidans, Acidianus brierleyi, Citrobacter, Clostridium, Cronobacter, Ferribacterium limneticum, Ferroplasma acidiphilum, Gallionella ferruginea, Leptospirillum ferrooxidans, L. ferriphilum, Ochrobactrum anthropi, Sulfolobus* sp., *Sulfobacillus thermosulfidooxidans, S. acidophilus, Thiobacillus denitrificans*, and *T. thioparus* (Schippers et al., 2010; Martins et al., 2011; Anawar, 2015). The survival and activities of these microbial communities are important in remediation AMTW.

The AMTW remediation with SRB is usually based on mixtures of salts and locally available organic substrates such as manures, sawdust, spent mushroom compost, sugarcane waste, wood chips, yeast extract and other carbon sources for bacteria metabolism. The optimization of these mixtures is important in order to achieve maximum metal and sulfate removal (Kefeni et al., 2017). The addition of organic matter and calcium carbonate helps to reduce the initial pH of AMTW that affects the growth of SRB. The mechanism of remediation involves the conversion of sulfate to hydrogen sulfide and conversion of organic matter to hydrogen carbonate. Heavy metal ions present then reacts with the hydrogen sulfide gas produced to form insoluble metal sulfide precipitates and the metals are removed through sulfide precipitation (Kefeni et al., 2017).

Zhang and Wang (2014), showed in a bioreactor experiment how dairy and chicken manure, and sawdust were used as organic carbon sources for heavy metal and sulfate removal by SRB (**Table 1**). Sulfate removal of 79, 64, and 50% were achieved using chicken and dairy manure, and sawdust, respectively, by SRB after 35 days of treatment. Sawdust showed poor performance compared to the manure as a carbon source to promote SRB growth due to low biodegradable fraction and low acidity (Zhang and Wang, 2014). Metals removed from the bioreactors were Cd, Cu, Fe, Mn, Ni and Zn. The results suggest that the growth of SRB can be promoted by continuous inputs of organic carbon for heavy metal and sulfate removal by SRB.

**Table 1:** Microbial remediation of acid mine tailings wastes.

Isolate	Source of acid mine waste	Carbon source amended with the medium of growth	pH	Sulfate removed (%)	Metals removed	Reference
Consortium of Acidophiles	Copper mine tailings	–	2.29	–	Cu, Fe, Zn	Bryan et al., 2006
*Desulfovibrio desulfuricans* and *Desulfomicrobium baculatum*	Synthetic mine waste	Ethanol	3–4	90	As, Cu, Fe, Ni, Zn	Sahinkaya et al., 2015
Sulfate-reducing bacteria	Synthetic acid mine drainage	Chicken manure Dairy manure Sawdust	3.0–3.5	79.04 64.78 50.27	Cd, Cu, Fe, Mn, Ni, Zn	Zhang and Wang, 2014
Immobilized sulfate reducing bacteria sludge granules	Synthetic acid mine drainage	–	2.8	80.2	Cd, Cu, Fe, Mn, Zn	Zhang and Wang, 2016
Consortium of sulfate reducing bacteria	Synthetic wastewater	–	6.0	67	Cu, Cr, Ni, Zn	Kieu et al., 2011
Sulfate reducing bacteria	Synthetic wastewater and acid mine from copper mine	Lactate	2.75	61	Cu, Fe, Mn	Bai et al., 2013
*Leptospirillum ferriphilum* and *Acidithiobacillus caldus*	Chalcopyrite mines	–	2.0	–	Cu, Fe	Zhou et al., 2009

Sahinkaya et al. (2015) assessed sulfate removal of As, Cu, Fe, Ni and Zn containing acid mine drainage in an up-flow sludge blanket reactor in an anaerobic condition. The bioreactor was operated for 500 days with ethanol as carbon and electron source (**Table 1**). Sulfate removal efficiency of 99% was achieved while metal removal was 98–100% for As and 99% for Cu, Fe, Ni and Zn. The SRB species identified in the column reactor were *Desulfovibrio desulfuricans* and *Desulfomicrobium baculatum*.

Sulfate-reducing bacteria have also been successfully applied on the industrial scale for the remediation of acid mine wastewaters. The two patented bioreactors in which SRB has been used are BioSulphide^®^, produced by BioteQ Environmental Technologies Inc., Canada and Thiopaq^®^, by Paques, The Netherlands (Kousi et al., 2015; Hussain et al., 2016). The BioSulphide^®^ technology has been successfully used to remove sulfate and heavy metals from acid mine drainage to produce high quality and marketable metal sulfides. The bioreactor involves two mechanism that works independently of each other: the biological and chemical components. The biological technique involves the reaction between elemental sulfur with an electron donor (acetic acid) in the presence of SRB under anaerobic condition to generate hydrogen sulfide. The SRB act as catalyst for the reaction to proceed kinetically at 25°C (Bratty et al., 2006).

(1) $$\begin{array}{c} {4S+CH}_{3}{COOH+2H}_{2}O\to {4H}_{2}{S+2CO}_{2} \end{array}$$

Hydrogen sulfide is continuously produced by removing the carbon (iv) oxide produced in the bioreactor. The reactor is a stirred tank with hydraulic retention time of several months. The H_2_S produced is passed through an agitated anaerobic contactor where targeted metals are precipitated as sulfides in the chemical process. The sulfides can then be dewatered in a conventional filtration unit. The filtration unit produces high grade metal sulfide that are further refined (Bratty et al., 2006; Littlejohn et al., 2015). Heavy metals targeted are typically As, Cd, Co, Cr, Cu, Hg, Mn, Mo, Ni, Pb, Se and Zn.

(2) $$\begin{array}{c} M^{2}{}^{+}{+H}_{2}S\to {\mathrm{MS}}_{2}^{–}{+2H}^{+} \end{array}$$

Thiopaq^®^ technology utilizes two different microbial population and procedure (Johnson and Hallberg, 2005). The first involves the generation of sulfide from sulfate by SRB and precipitation of metal sulfides while the second process involves the conversion of excess H_2_S produced to elemental sulfur by sulfide-oxidizing bacteria (Johnson and Hallberg, 2005).

(3) $$\begin{array}{c} H_{2}{\mathrm{SO}}_{4}{+4H}_{2}+½O_{2}\to S^{–}{+5H}_{2}O \end{array}$$

Metal of interest or those present in the waste stream can then be precipitated out as metal sulfide. Metal recovery is based on the solubility of metal sulfides at different pH values.

These bioreactors are proving to be effective in metal removal and/or recovery and in environmental pollution control. They are cost effective and enables the reduction, and elimination in some cases, of sludges that otherwise required expensive disposal through the production of high quality water for discharge into the environment. These technologies also reduced the inhibition of SRB by dissolved metals and allows optimum operation of the bioreactors (Johnson and Hallberg, 2005; Bratty et al., 2006; Littlejohn et al., 2015).

### Selection of Resistant SRB and Their Metabolic Properties

The microbial diversity found in acid mine sites includes organisms of the primary domains of bacteria, archaea and eukarya (fungi and algae). The bacteria diversity in acid mine belong to the phyla *Acidobacteria, Actinobacteria, Bacteroidetes, Firmicutes, Nitrospirae*, the alpha, beta and gamma classes of the phylum *Proteobacteria* (the most widely distributed phylum in acidic mine), and some archaea taxa (Johnson, 2012; Méndez-García et al., 2015). The presence of these organisms at acid mine sites are restricted to *Deltaproteobacteria* and *Firmicutes* (Méndez-García et al., 2015). These organisms exhibit a wide range of metabolic activities by utilizing solar or chemical energy. They utilize organic or inorganic carbon (such as ferrous iron, hydrogen, short-chained fatty acids, and reduced sulfur) as their sole carbon source and also grow under aerobic and anaerobic conditions (Johnson and Hallberg, 2009; Johnson, 2012). Méndez-García et al. (2015) described SRB as chemolithotrophic or chemoorganotrophic organisms that utilize sulfate as the terminal electron acceptor. They couple the oxidation of organic substrates to reduction of sulfate generating hydrogen sulfide as the major end-product.

Due to relatively low concentration of organic matter in AMTW, an external source of carbon and an electron donor are required for SRB and bioreactor effectiveness. The choice of the external carbon source is determined by the following factors: the suitability of the carbon substrate for a specific application, the substrate utilization potential of the SRB, the volume of sulfate to be reduced, substrate cost per unit hydrogen sulfide produced and by-product from incomplete substrate utilization (Kaksonen and Puhakka, 2007). Combination of more than one carbon substrate increases sulfate reduction. Increase or decrease in substrate mixture have a major impact on SRB efficiency and is also important in reducing the adverse effects of toxic metals by acid buffering and adsorption (Kefeni et al., 2017).

Various intermediate products from anaerobic degradation of complex organic compounds such as carboxylic acids (acetate, butyrate, fumarate, malate), amino acids (alanine, glycine, serine), alcohols (ethanol, methanol), hydrogen, methanethiol, some sugars (fructose, glucose) and aromatic compounds (ethylbenzene, benzoate, phenol and toluene) are oxidized by SRB. These organisms use the Calvin-Benson-Bassham cycle to obtain cellular carbon and the enzyme that plays a key role in this pathway is ribulose bisphosphate carboxylase/oxygenase (Johnson and Hallberg, 2009). They oxidize organic substrate either by complete oxidation producing carbon dioxide or acetate in incomplete oxidation (Kaksonen and Puhakka, 2007; Muyzer and Stams, 2008). The lack of acetyl-CoA oxidation mechanism is responsible for the incomplete oxidation process. Acidic waste is neutralized by bicarbonates produced from acetate oxidation. This is an important step in the treatment process of AMTW. Acetate can also be used as a source of carbon by some incomplete oxidizers in the presence of hydrogen as electron donor (Kaksonen and Puhakka, 2007). According to Muyzer and Stams (2008), SRB have not been able to grow directly on substrates such as cellulose, fats, nucleic acids, polymeric organic compounds, proteins and starch, but they depend on other microorganisms that degrade these substrates and ferment them to products that can be used by SRB.

Sulfate reducing bacteria can also grow by the splitting of thiosulfate, sulfite and sulfur, which results in the formation of sulfate and sulfide (Muyzer and Stams, 2008). The process of sulfur metabolism involves the requirement of ATP to reduce sulfate by two key enzymes adenylylsulfate reductase and bisulfite reductase. The process involves sulfate activation and reduction of sulfate to sulfite, sulfite reduction to sulfide, and elemental sulfur reduction (Barton and Fauque, 2009). Sulfur-oxidizing acidophiles are also able to couple the oxidation of sulfur to reduction of iron which has been observed in *At. thiooxidans* and *At. ferrooxidans* (Johnson and Hallberg, 2009). During the oxidation of reduced inorganic sulfur through sulfite production, sulfide is oxidized by a sulfide/quinone oxidoreductase, which transfers electrons to ubiquinone and generates sulfur. The sulfur generated can be oxidized to sulfite by the periplasmic sulfur dioxygenase. The sulfite produced is then oxidized to sulfate by the enzyme sulfite oxidoreductase or through the action of adenosine phosphosulfate reductase (Rohwerder and Sand, 2003; Méndez-García et al., 2015). The Sulfate produced is incorporated into the amino acids methionine and cysteine, iron-sulfur centers and other metabolites. SRB can also grow in the absence of sulfur and can form syntrophic association with methanogens or other hydrogen-scavengers (Muyzer and Stams, 2008; Plugge et al., 2011). This enhances their survival in the environment when electron acceptors become depleted.

Most SRB grow optimally between pH 6 and 8 (Sánchez-Andrea et al., 2015). Beyond this pH range, there is a decline in microbial sulfate reduction rate and reduction in metal removal capacity. Acidic pH increases metal sulfide solubility (Sheoran et al., 2010). Some SRB species can tolerate pH values of between 5 and 9.5. Some acidophilic and acidotolerant SRB have been isolated on solid growth medium at pH 3.6 (Sen and Johnson, 1999). The introduction of acid tolerant SRB into bioreactor systems has improve performance and led to the development of innovative bioreactor systems for recovery of metals (Sheoran et al., 2010). For acidophilic organisms to survive in this environment, they need to possess two physiological traits essential for survival. They must possess unusual ability to prevent the entry of proton into the cell cytoplasm, even when presented with a high inward gradient. Acidophilic SRB make use of pH gradients to generate ATP using membrane-bound ATPases. The second feature is the ability to generate positive membrane potentials which is achieved by active influx of cations. This gives a level of protection against many positively charged ions (Johnson and Hallberg, 2009).

Sulfate reducing bacteria are mostly mesophilic, but also include some thermophilic and psychrophilic species. These organisms can also tolerate temperatures from below 5° to 75°C. Temperature affects the rate of microbial processes with decrease in microbial processes as temperature decreases (Sheoran et al., 2010). Increase in temperature may help SRB to outcompete methanogens in AMTW. High temperature increases metabolic rate and reduces toxic hydrogen sulfide solubility (Kaksonen and Puhakka, 2007).

### Limitations of Remediation of Acid Mine Wastes by Sulfate-Reducing Bacteria

One of the limitations of the application of SRB in the treatment of AMTW is the sensitivity of SRB to low pH, which affects the growth of microbes and impairs bioreactor performance. The pH directly influences the microbial communities which thrive in the reactors and also directly affects the quality and size of the metal sulfides (Sánchez-Andrea et al., 2014; Kefeni et al., 2017). According to Zhang and Wang (2016), the optimum pH for growth of SRB is around pH 6–8 and many are sensitive to low pH with most SRB being inhibited at pH 5.5. This challenge can be overcome through the addition of alkaline materials such as calcium carbonate, caustic soda, lime and soda ash. This helps to increase the pH of AMTW and also improve the performance of SRB by serving as a source of nutrients for these organisms (Kefeni et al., 2017). Most reactors also need to avoid direct contact between the acidic waste stream and SRB. The use of acidophilic and acidotolerant SRB in bioreactors has also improved the operation of these reactors.

In addition, AMTW treatment requires regular supply of biomass feed to the reactors to enhance the microbial activity due to low concentration of organic matter. However, the supply of organic substrate and alkaline materials increases the operational cost of remediation. The choice of suitable organic substrate is important for efficient process and economical advantage (Sheoran et al., 2010). The availability and nature (biodegradable) of the substrate is equally important.

Furthermore, bioreactor design and installation are sometimes expensive, the stability and fate of accumulating deposits within bioreactors are unknown, and their performance is less predictable than chemical treatment systems. Equally, these technologies may not be feasible in some geographical locations which may ultimately affect efficiency of bioreactors in sulfate and metal recovery as well as limited applications as full-scale systems. This is as a result of significant variability in the chemistry (pH, metal loading) and flow rates of AMTW (Johnson and Hallberg, 2005; Sánchez-Andrea et al., 2014).

Another drawback of SRB reactors is metal toxicity to SRB organisms. High concentration of metal cations in AMTW may be toxic or inhibitory to the activities of SRB communities. Their survival is important for sulfide formation and metal recovery (Zhang and Wang, 2016). Metal toxicity depends on other parameters that includes type and concentration of metal, quantity of biomass, source of carbon, pH, temperature, sulfate concentration, and presence and concentration of iron and complexing compounds. The pH and the temperature of the environment affect the physiological state of SRB, thus impacting on their metal tolerance. Binding character and charge of metals is also affected by pH. In the presence of hydroxyl ion and other inorganic anions, metals complexes are formed with different toxicities and binding characters compared to non-complexed metals (Kaksonen and Puhakka, 2007).

Hence the need to engage more efficient processes for higher removal rate of sulfate and heavy metals and the need to control microorganism by means of coordinated approach. By and large, understanding of the basics of microbial groups in key processes will be significant in handling important environmental challenges (Verstraete and De Vrieze, 2017).

## Engineered SRB Consortium to the Rescue

Engineered microbial consortia could be employed in industrial bioreactors to enhance metal and sulfate elimination. These engineered consortia might be used to culture many of the yet uncultured microbes that are abundant in acid mine, their characteristics and elucidate their functional roles (Brune and Bayer, 2012).

Microbial consortia play a crucial role in remediation and are known to accelerate complicated tasks that allow the survival of microorganisms in hostile environments such as the AMTW and can be more robust to environmental fluctuations (Brune and Bayer, 2012). They have been successfully applied to real world problems and facilitate complex functions involving inter-species interactions that enable their survival in acid mine environment (Keller and Surette, 2006). They are involved in syntrophic (feeding together) degradation of complex substances that permits complete metabolic reactions from two or more organisms without which energy cannot be gained without the collaboration of the other (Zhou et al., 2011). Simultaneous removal of toxic metals and sulfate from contaminated environment can be achieved using the combination of specific properties of these organisms and interacting metals. These organisms are a rich source of genetic information which can be used to create modified microbiomes that concentrate metals and sulfate (Dunbar, 2017). These organisms also offer a variety of resistance mechanism that enhances remediation. Efficient application of these organisms requires highly resistant microorganisms with natural or engineered degradation pathway.

The mechanism involved in bioleaching of metals and adaptation of iron- and sulfur oxidizing microbes to the harsh acid mine environment can be exploited in microbial consortium remediation of AMTW (Latorre et al., 2016). Brune and Bayer (2012) proposed that substrate utilization among naturally occurring consortia of autotrophic iron-oxidizing and sulfur-oxidizing microorganisms is either symbiotic, potentially mutualistic or synergetic. The synergistic role between different strains can be useful for better balancing of the metabolism of iron and sulfur. However, the functions of the bacterial consortium is still not fully understood (Latorre et al., 2016).

Synthetic biology to design microbial consortia was suggested by Brenner et al. (2008). They also suggested that cell signaling and communication is the first step in constructing engineered consortium. Communication allows for division of labor among the individuals or populations which enables them to perform the complex task of degradation (Hays et al., 2015). Division of labor also confer additive benefits on the participating organism. Communication involves the exchange of dedicated signal molecules within or between single populations which can be driven by social characteristics or based on metabolic properties. The behavior is coordinated with the exchange of small peptides (Gram positive organisms) and acyl-homoserine lactone (acyl-HSL) signaling molecules (Gram negative organisms) in bacteria (Brenner et al., 2008).

Extracellular polymeric substances (EPS) production by bioleaching model organism *At. ferrooxidans* suggest that communication play a major role in the formation of biofilm, which is essential for contact leaching mechanism (McDougald et al., 2012). This organism secretes and respond to compounds of the acyl homoserine lactones (AHLs) family utilized as a part of auto-inducer1 (AI-1) type quorum sensing system and additionally the c-di-GMP signaling pathway also used in quorum sensing (Keller and Surette, 2006; Brune and Bayer, 2012). It might be possible to alter the AHL- and c-di-GMP levels to enhance the organism’s attachment to ore particles which can improve leaching process in *At. ferrooxidans* through synthetic biology (Brune and Bayer, 2012). In like manner, inter-population communication can also include exchange of chemicals involved in metabolism and growth.

Brune and Bayer (2012) propose that synthetic biology can change the behavior and performance of microbial consortia associated with metal extraction and metal contamination. Synthetic consortia can help to cultivate and make use of the unculturable microorganisms to explain the functions of many unknown genes of relevance to microbial consortia. Microbial communities in and near acid mines are therefore a rich source of genetic information which could be used to create synthetic or modified microbiomes that concentrate metals and remove sulfate. Understanding of the survival of acidophiles in AMTW environments is important to have a better approach for metal biomining and also for bioremediation of acid mines and to enhance and generate new biotechnological cleaning techniques (Jerez, 2017). In industrial applications, engineered consortia may allow for more complex undertakings.

Latorre et al. (2016) evaluated a consortium of five bacteria (*At. ferrooxidans* Wenelen, *At. thiooxidans* Licanantay, *Acidiphilium multivorum* Yenapatur, *Leptospirillum ferriphilum* Pañiwe, and *Sulfobacillus thermosulfidooxidans* Cutipay) for their bioleaching potential of copper based on tolerance to metal and oxidation activity. Two of the organisms, *L. ferriphilum* and *At. thiooxidans* were reported to have higher copper recovery efficiency and the ability to oxidize iron and reduced inorganic sulfur compound. The consortium confers special ability to leach copper sulfide ores and heavy metals (arsenic and copper) by these bacteria. The genome of the five organisms reveals information about specific roles each play in the consortium which can be used in the design of bioleaching plant and how interventions can be made directly in bioreactors (Latorre et al., 2016).

Once an initial consortium is developed, evolution theory can possibly be used to elucidate novel species interactions which can result in enhanced microbial consortium productivity and stability (Brune and Bayer, 2012). However, the field of synthetic biology is evolving and more systematic and integrated efforts are required for next generation technologies. The field of single cell genomics and metagenomics will be a useful tool for understanding the genetic diversity of yet to be cultured organisms in various environment which will also lend support to the understanding of functional roles of these organisms in such environment (Zhou et al., 2011).

The application of these organisms for treatment of AMTW is a suitable approach to remove persistent pollutant in the environment. While the synthetic biology keeps producing a number of engineered consortium for the purpose of bioremediation, there are concerns about their uncertain effects on the ecosystem (Lorenzo, 2017).

## Conclusion

Poor waste management and rehabilitation, coupled with an emphasis on production over environmental impacts, which is related to challenges of varying degrees of difficulty, has caused substantial environmental impacts in the mining industry. Reducing the environmental and health impact of mine wastes requires the development of an appropriate remediation strategy. Through the development of best management practices with sustainable development in mind, environmental threats from mining and mineral processing can be reduced. Overall, this review has shown that bioremediation is an effective technique and environmentally sustainable approach for AMTW pollution reduction. However, there is need for ongoing research to identify suitable sulfate-reducing organisms and environmental conditions to meet the economical target of bioremediation. The design of engineered microbial consortium can enhance remediation beyond productivities observed in natural consortium.

## Author Contributions

AA was responsible for data collection and wrote the first draft of the manuscript. OO was responsible for graphic design of the manuscript and read the first draft of the manuscript. OOB was responsible for conception and design of the manuscript and revising it critically.

## Conflict of Interest Statement

The authors declare that the research was conducted in the absence of any commercial or financial relationships that could be construed as a potential conflict of interest. The reviewer VG and handling Editor declared their shared affiliation.
